# Natural experiments in the built environment: evaluating impacts on health

**DOI:** 10.24095/hpcdp.46.3.01

**Published:** 2026-03

**Authors:** Adrian Bauman, Melanie Crane

**Affiliations:** 1 Prevention Research Collaboration, Sydney School of Public Health, Charles Perkins Centre, The University of Sydney, Camperdown, New South Wales, Australia; 2 Health Promotion, Centre for Population Health, Western Sydney Local Health District, Cumberland Hospital Campus, North Parramatta, New South Wales, Australia

Our understanding of how the built environment affects health outcomes has improved in recent decades through the use of natural experiments. Natural experiments are observational studies of naturally occurring phenomena or events that show variation, in which exposure to different levels or types of exposure are assessed in relation to defined health outcomes.^1^,^2^ Natural experiment methods are particularly useful for evaluating the effects of built environment changes.

Natural experiments can be new policies, infrastructure or interventions that researchers may neither be able to, or should (for ethical or practical reasons), control or manipulate.^1^ This means that there is generally no input into the intervention design a priori. The agencies or actors implementing such interventions can be governments, municipalities or non-governmental organizations. Natural experiments can also be naturally occurring events such as catastrophic weather events, major economic downturns or societal changes such as the COVID-19 pandemic.^3^ Published natural experiment interventions are typically characterized by changes in policies or regulations related to the environment or in health service provision, which cannot easily be evaluated using traditional evidence-generating research designs.^4^

Natural experiments are important in evaluating community-level interventions that are relevant to population health and well-being. A broader strength of natural experiments is their ability to assess complex interventions at scale, including obesity-prevention policies such as food labelling and restricting advertising of or taxing unhealthy products, among other examples.^5^ Natural experiments are useful where controlled trial designs are not amenable, such as the effects of alcohol policies or fluoridation of water.^6^,^7^

Many built environment interventions are outside of the control of health researchers, which results in limited evidence on their health outcomes.^8^ Natural experiments enable health researchers to work alongside planners, transportation and other city sectors to investigate the effects of planned or unplanned urban interventions. A recent systematic evaluation of natural experiments provides valuable evidence in summarizing the effects of the built environment.^9^ Still, natural experiments cannot always provide strong causal evidence, given the complexities of built environment interventions. This means that there is a risk of bias in study designs;^10^ however, this is outweighed by the flexibility natural experiments provide in complex evaluations.

There have been several iterations of best practice guidelines for natural experiments. In the most recent, in 2025, researchers with the United Kingdom’s National Institute for Health and Care Research and the Medical Research Council developed a framework for quality evidence in natural experiment design.^1^ The starting point for natural experiments is to determine if methods can be applied to usefully understand or explain the changes induced by the naturally occurring phenomenon.^11^ Natural experiment evaluations utilize diverse methods, including quantitative designs, qualitative research, economic evaluations and routinely collected health or social indicator data.^1^ The most frequently used quantitative research designs use repeat cross-sectional surveys, quasi-experimental designs, interrupted timeseries designs and difference-in-difference methods.^1^ Qualitative methods may assist in understanding the intervention context and the actors, partners and affected groups involved and in explaining potential mechanisms of change. It is usual for natural experiments to require complex system evaluation methods as multiple components of interventions are often delivered across diverse settings.^4^

Given the intersectoral nature of built environment interventions, it is often necessary to use natural experiment methods in their evaluations. Examples include assessing the effects of introducing healthy city policies or housing redevelopment policies within an urban area on community well-being, dietary habits or physical activity.^12^,^13^ Sometimes natural experiments can assess interventions across communities, such as in the evaluation of the 19 rural communities in the Alberta Healthy Communities Approach by Gillies et al.,^14^ which showed positive effects of built environment changes and support for healthy eating and physical activity programs. Not all natural experiments show positive results, as Belon and colleagues’ study of urban infrastructure regeneration planning in Alberta demonstrates.^15^ This study highlights the challenges of defining exposure, determining when data are collected, where study participants are selected and the difficulty of finding representative comparison groups. Sometimes evaluation methods from both implementation science and from scale-up evaluation^16^ are useful to assess the reach, adoption and adaptation of community-wide interventions.

Another Canadian study looked at cycling infrastructure in Montral, Quebec, and evaluated the intervention using biennial national population health surveys and geographical information systems data. Prince et al. showed that decreased distance to and increased length of bike paths were related to increased usage over time.^17^ Similarly, natural experiments evaluating public open spaces revealed that access to open spaces was partly responsible for maintaining social connections during the COVID-19 pandemic lockdown.^18^ Many studies that examined park design improvements and outdoor exercise facilities have included serial time series measurements and compared differences to parks that had not undergone changes as controls, and demonstrated increased usage and increased diversity of park users.^19^ More broadly, a systematic review of natural experiments of real-world built environment interventions across Canada conducted by Prince et al. revealed positive effects of walkable community interventions, cycling–pedestrian infrastructure and bike-share schemes; however, less evidence was apparent from school or daycare infrastructure improvements or new bus route interventions.^9^

These recent examples demonstrate the kinds of interventions that require natural experiments for their evaluation. Future opportunities related to the built environment could include contributions from citizen science and individual wearable-technology data, using geographic information systems and movement cameras. Opportunities should also be taken to strengthen networks between public health research, practice and policy, particularly across city sectors driving health outcomes.^20^ Opposition may come from the research community, guided by the biomedical hierarchy of evidence, which may consider the evidence from natural experiments as not “reasonable causal evidence.” This opposition has necessitated several attempts to define the attributes of good quality natural experiment research,^1^,^4^ and it is hoped that policy-makers will appreciate when natural experiment have been rigorous enough to provide policy relevant advice.
